# Post-Radiotherapy PET Image Outcome Prediction by Deep Learning Under Biological Model Guidance: A Feasibility Study of Oropharyngeal Cancer Application

**DOI:** 10.3389/fonc.2022.895544

**Published:** 2022-05-13

**Authors:** Hangjie Ji, Kyle Lafata, Yvonne Mowery, David Brizel, Andrea L. Bertozzi, Fang-Fang Yin, Chunhao Wang

**Affiliations:** ^1^ Department of Mathematics, North Carolina State University, Raleigh, NC, United States; ^2^ Department of Radiation Oncology, Duke University Medical Center, Durham, NC, United States; ^3^ Department of Radiology, Duke University Medical Center, Durham, NC, United States; ^4^ Department of Electrical and Computer Engineering, Duke University, Durham, NC, United States; ^5^ Mechanical and Aerospace Engineering Department, University of California, Los Angeles, Los Angeles, CA, United States; ^6^ Department of Mathematics, University of California, Los Angeles, Los Angeles, CA, United States

**Keywords:** biological modeling, deep learning, image outcome prediction, radiotherapy, ^18^FDG-PET

## Abstract

**Purpose:**

To develop a method of biologically guided deep learning for post-radiation ^18^FDG-PET image outcome prediction based on pre-radiation images and radiotherapy dose information.

**Methods:**

Based on the classic reaction–diffusion mechanism, a novel biological model was proposed using a partial differential equation that incorporates spatial radiation dose distribution as a patient-specific treatment information variable. A 7-layer encoder–decoder-based convolutional neural network (CNN) was designed and trained to learn the proposed biological model. As such, the model could generate post-radiation ^18^FDG-PET image outcome predictions with breakdown biological components for enhanced explainability. The proposed method was developed using 64 oropharyngeal patients with paired ^18^FDG-PET studies before and after 20-Gy delivery (2 Gy/day fraction) by intensity-modulated radiotherapy (IMRT). In a two-branch deep learning execution, the proposed CNN learns specific terms in the biological model from paired ^18^FDG-PET images and spatial dose distribution in one branch, and the biological model generates post-20-Gy ^18^FDG-PET image prediction in the other branch. As in 2D execution, 718/233/230 axial slices from 38/13/13 patients were used for training/validation/independent test. The prediction image results in test cases were compared with the ground-truth results quantitatively.

**Results:**

The proposed method successfully generated post-20-Gy ^18^FDG-PET image outcome prediction with breakdown illustrations of biological model components. Standardized uptake value (SUV) mean values in ^18^FDG high-uptake regions of predicted images (2.45 ± 0.25) were similar to ground-truth results (2.51 ± 0.33). In 2D-based Gamma analysis, the median/mean Gamma Index (<1) passing rate of test images was 96.5%/92.8% using the 5%/5 mm criterion; such result was improved to 99.9%/99.6% when 10%/10 mm was adopted.

**Conclusion:**

The developed biologically guided deep learning method achieved post-20-Gy ^18^FDG-PET image outcome predictions in good agreement with ground-truth results. With the breakdown biological modeling components, the outcome image predictions could be used in adaptive radiotherapy decision-making to optimize personalized plans for the best outcome in the future.

## Introduction

Radiotherapy is a central component of the standard of care for many cancers. In the current era of image-guided radiotherapy (IGRT), medical imaging plays a critical role in radiotherapy practice regarding patient assessment, treatment volume definition, on-board patient positioning, and outcome assessment (1). In particular, imaging-based radiotherapy outcome assessment can capture early therapeutic responses for adaptive therapy to enhance radiotherapy efficacy (2). In addition, long-term therapeutic outcomes from image-based analysis provide useful information in treatment intervention of each patient towards optimized cancer care (3). Thus, medical imaging analysis for radiotherapy outcome assessment has become an irreplaceable component in precision medicine.

Technologies of medical imaging analysis have revolutionized image-based radiotherapy outcome reporting. Radiographic assessment of post-radiotherapy tumor morphological changes (i.e., Response Evaluation Criteria in Solid Tumors [RECIST]) was standardized to describe the response to therapy (4). Functional imaging modalities have now shifted outcome analysis from morphological description to physiological characterization. PET tracks the *in vivo* radioactive tracer distribution, for example, estimating glucose metabolism (^18^F-FDG) or measuring tissue hypoxia (^18^FMISO) (5). MR functional imaging, including dynamic contrast-enhanced MRI (DCE-MRI), diffusion-weighted MRI (DWI), and diffusion tensor MRI (DTI), can measure tissue properties such as blood volume/perfusion (6), cellular density (7), and cell movement direction (8). To non-invasively quantify *in vivo* physiology, functional imaging relies on mathematical models to extract quantitative parameters from phenotype image data. These mathematical models, which are often referred to as mechanism-based models, describe complex physiological processes using basic biological theories and fundamental laws in physical/chemical interactions (9, 10). The derived parameters of mechanism-based models can serve as surrogates of individual physiology functions to facilitate developing a personalized therapeutic approach.

Treatment response assessment using functional imaging is often reported as posttreatment changes relative to pretreatment baseline values. Image-based treatment outcome prediction, i.e., forecasting posttreatment image volumes before treatment initiation, has become an emerging topic in clinical oncology (11). The potential clinical application of image-based treatment outcome prediction in radiotherapy is conceptually promising: given an individual’s pre-radiotherapy image, post-radiotherapy image predictions could be available at the treatment planning stage. Guided by these predictions, clinicians could simulate alternative treatment plans, such as target delineation revision and plan parameter tuning (beam angle, energy selection, etc.), for normal tissue sparing and could select a plan that predicts improved response to radiotherapy. This scenario can be applied to adaptive radiotherapy: the predicted intra-treatment images can be used to determine whether a revised radiotherapy plan would be advantageous. Additionally, when new intra-treatment image data are collected, the updated predictions can guide the adaptive planning strategy for optimal radiotherapy outcomes for individual patients (10). Driven by the rapid growth of computation power, deep learning techniques have recently become a practical approach for image-based treatment outcome prediction (12–14). However, few investigators have reported functional image outcome prediction in radiotherapy applications. Aside from the colossal computational workload due to image dimension requirement, the current mechanism-based models focus on spatial decoding of physiology within an image volume; for outcome prediction, a mechanism-based model must incorporate patient-specific treatment information to simulate spatiotemporal physiology evolution during a treatment course. Although pilot studies have reported the feasibility of post-radiotherapy functional image outcome prediction using treatment information (15), the adopted deep learning network ignored the biophysical modeling and generated its prediction *via* a “black box”; thus, the achieved prediction was reported at a fixed time point without any biological interpretation about how radiation dose affects the outcome. Radiotherapy outcome prediction with breakdowns from biological modeling is an unmet need.

In this work, we design a biologically guided deep learning framework for intra-treatment ^18^FDG-PET image outcome prediction in response to oropharyngeal cancer intensity-modulated radiotherapy (IMRT). Based on the classic reaction–diffusion mechanism in disease progression, we propose a novel partial differential equation (PDE) as a biological model that incorporates spatial radiation dose distribution as a patient-specific treatment information variable. An encoder–decoder-based convolutional neural network (CNN) is designed and trained to learn the proposed model, which governs the dynamics of tissue response to radiotherapy. Thus, with the explainability of the biological model, the developed deep learning model can generate post-radiotherapy ^18^FDG-PET image outcome predictions with breakdown biological components.

## Materials and Methods

### Biological Modeling

We hypothesize that the standardized uptake value (SUV) change in ^18^FDG-PET in response to radiation can be described in a reaction–diffusion system, which represents a family of mathematical models widely used in describing pattern formations and evolving densities in physical, ecological, and biological systems (16). In the context of modeling tumor growth and therapeutic response dynamics, reaction–diffusion models have been applied to both preclinical and clinical works (9, 17, 18). Disease progression, in general, can be summarized by Eq. (1), which describes the malignancy proliferation (reaction) and spread (diffusion) (10):


(1) $$U_{t}=\alpha \Delta U+\beta U$$

where *U* is the spatial distribution of disease (i.e., SUV intensity distribution in this work) and $U_{t}=\frac{\partial U}{\partial t}$ is the time derivative term describing the change of *U* in time. The term $\alpha \Delta U=\alpha (\frac{\partial^{2}U}{\partial x^{2}}+\frac{\partial^{2}U}{\partial y^{2}})$ describes the spreading of abnormal cell activities, where *α* > 0 is the diffusion coefficient. The linear term *βU* represents the proliferation of localized malignancy. To incorporate tissue response to radiotherapy in the model in Eq. (2), we propose a new response term for the dose-induced changes of *U*,


(2) $$U_{t}=\alpha \Delta U+\beta U+F\left(DU\right)$$

where F(*DU*) is an 𝒩 unknown operator that depicts *U*'s local response to radiotherapy. Here we assume that the response term depends on the product of *U* and the radiotherapy plan’s spatial dose distribution *D*. We also assume that the operator *F* depends on *DU* as the tissue response to cell killing from localized high radiation (10), and we will use a CNN to learn this operator. Thus, Eq. (2) is the core time-dependent PDE that models the post-radiotherapy biological response of abnormal tissue metabolism as SUV intensity (i.e., *U*) evolves in time.

### Deep Learning Design

Formally, our problem is defined as follows: given a set of pre- and post-radiation ^18^FDG-PET image pairs ${\left\{\left(U_{k}^{pre},U_{k}^{post}\right)\right\}}_{k=1,2,\ldots m}$ and the imposed radiation dose distribution images { *D*_*k*_ }_*k*=1,2,…*m*_ where *m* is the total number of image pairs, our goal is to learn the unknown response operator *F* and coefficients α, β in the model in Eq. (2) with the collected data of the form ${\left\{\left(U_{k}^{pre},U_{k}^{post},D_{k}\right)\right\}}_{k=1,2,\;\ldots m}$ . Accordingly the learned model can predict a post-radiation ^18^FDG-PET image $U_{k}^{post}$ given the pre-radiation image $U_{k}^{pre}$ and the associated spatial dose distribution *D_k._* In addition, since the learned model describes the evolution dynamics of *U_k_* between the two states $U_{k}^{pre}$ and $U_{k}^{post}$ frames of *U_k_* between $U_{k}^{pre}$ and $U_{k}^{post}$ can be simulated to study the intermediate stages of disease progression.

While a large body of work has focused on solving reaction–diffusion models like Eq. (2), i.e., finding *U* based on known coefficients and operators, little research has been devoted to the inverse problem of learning the model’s coefficients and operators from observed *U* data. The numerical treatments of the inverse problem are typically complicated, as the observed data usually cannot provide sufficient information to determine a unique model, and regularizations are needed to produce meaningful model estimates. As such, we propose a deep neural network framework to learn the model in Eq. (2) from ^18^FDG-PET images taken before and after radiation. Applying the forward Euler method on the PDE in Eq. (2), we obtain the discretized update rule:


(3) $$U^{n+1}=U^{n}+h\alpha \Delta U^{n}+h\beta U^{n}+hF\left(DU^{n}\right)$$

where *h* is the time step, *U^n^* is the approximate solution of the state *U* at the time *t_n_* =*nh*, and the Lssssaplacian operator Δ can be approximated by a discrete operator $D_{xy}^{2}$ represented by a nine-point refined stencil (19):


(4) $$D_{xy}^{2}=\left(\begin{array}{ccc} 1/4 & 1/2 & 1/4\\ 1/2 & -3 & 1/2\\ 1/4 & 1/2 & 1/4 \end{array}\right)$$

A deep neural network N_*F*_ is designed to approximate the response operator F:


(5) $$N_{F}:\psi \to N_{F}\left(\psi ;\;\theta \right)$$

where θ represents the free parameters. For simplicity, we assume that the operator *F* only depends on, *ψ*=*D U*, the product of the dose distribution *D* and the ^18^FDG-PET image state variable *U*. A diffusion–proliferation operator *G* is used to combine both the diffusion and proliferation terms with undetermined coefficients *α* and *β*s


(6) $$𝒢\left(U^{n}\right)=\alpha D_{xy}^{2}U^{n}+\beta U^{n}$$

Given a group of three images consisting of the initial state ^18^FDG-PET image $U_{k}^{0}=U_{k}^{pre}$ at *t* = 0 prior to radiation, dose distribution map *D_k_*, and the ground-truth final state ^18^FDG-PET image $U_{k}^{post}$ at *t* = *T* (post-radiation), from Eqs. (3)–(6), we obtain the intermediate states $U_{k}^{n+1}$ by 


(7) $$U_{k}^{n+1}=U_{k}^{n}+hG\left(U_{k}^{n}\right)+hN_{F}\left(D_{k}\circ U_{k}^{n};\theta \right)$$

for *n* = 0,1, …, *N_t_* − 1. Here, $D_{k}\circ U_{k}^{n}$ represents the element-wise product of the dose distribution map *D_k_* and the ^18^FDG-PET image $U_{k}^{n}$ at the time step *t_n_*, *N_t_* is the total number of steps, and the step size *h*=T/*N*_t._ As a feasibility study, we consider the final time *T* = 1 and set the number of steps *N_t_* = 4 in this work.

The similarity between the predicted post-radiation ^18^FDG-PET image $U_{k}^{N_{t}}$ and the associated ground-truth image $U_{k}^{post}$ is defined based on the *l*_2_ norm loss function:


(8) $$L\left(\theta \right)=\frac{1}{m}\sum_{k=1}^{m}{∥U_{k}^{N_{t}}-U_{k}^{post}∥}_{2}^{2}$$

where *m* is the number of samples. By minimixing *ℒ*(*θ*), the deep neural network can learn the weights *θ* that characterize the response operator F and the undetermined coefficients *α* and *β*.

**Figure 1:**
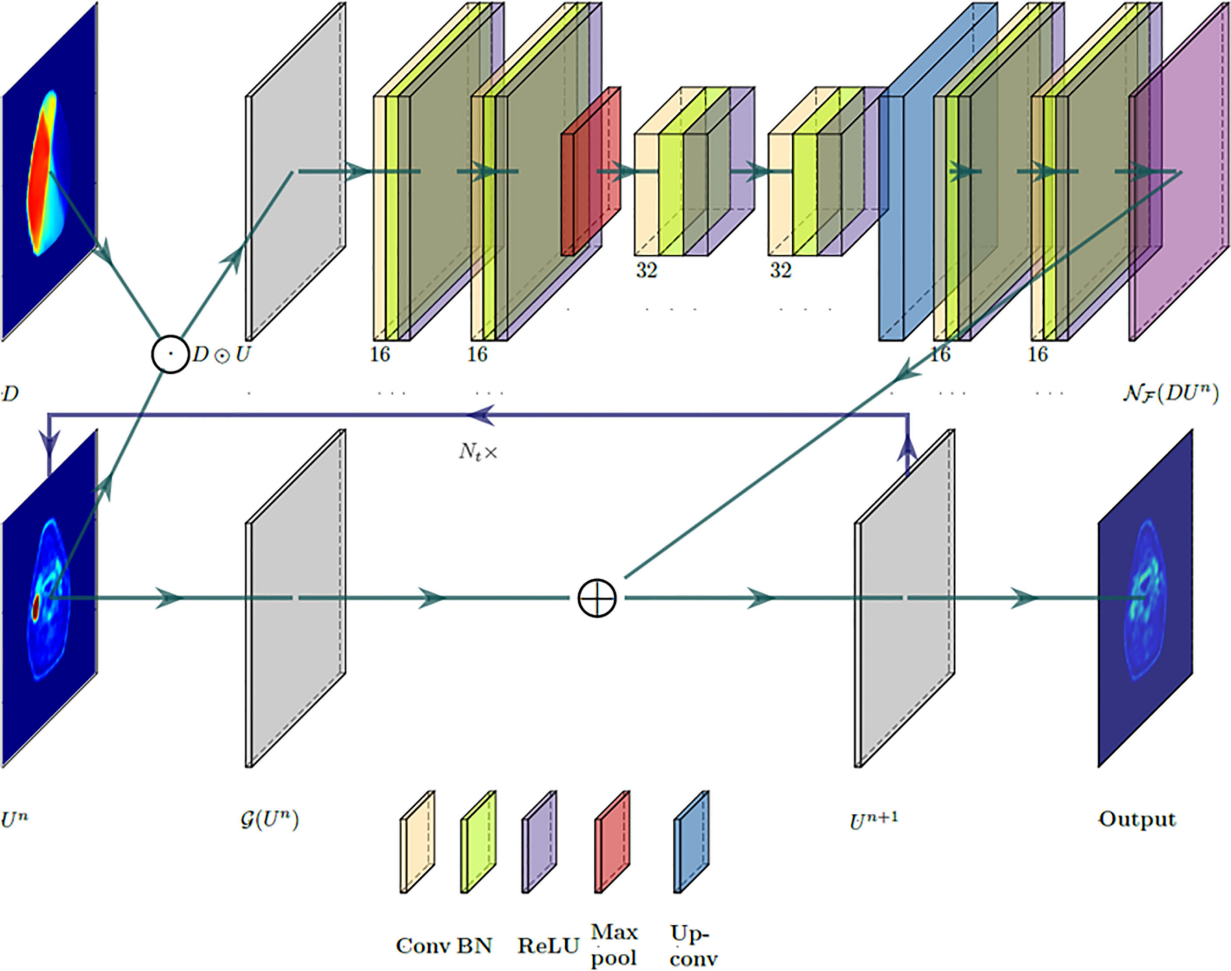
A partial differential equation (PDE)-informed deep neural network design. Layers are color-coded by operations with associated feature numbers.

**Figure 1** illustrates the designed deep neural network architecture. The network’s input space is composed of pre-radiation ^18^FDG-PET image *U^pre^* and planned dose distribution map *D* as a set. The network is split into two branches: one that uses a CNN to learn the response operator N_F_(*D U* ^*n*^) and the other one with only two trainable parameters to apply the diffusion–proliferation operator G [in Eq. (6)] on *U^n^*. Specifically, the second branch of the network architecture mimics the traditional finite difference method and applies the discrete Laplacian operator and the linear operator on *U^n^* with predicted *α* and *β*. Both branches are then merged by the rule in Eq. (7), which feeds the output *U^n^* ^+1^ forward to the next cycle. This process is then repeated for *N_t_* time steps to generate a predicted post-radiation ^18^FDG-PET image, which will be compared against the ground-truth post-radiation ^18^FDG-PET image.

The branch that learns the response operator N_F_(*D U* ^*n*^) consist of a total of 7 convolutional layers and is built upon U-Net’s encoder–decoder architecture (20). The architecture consists of a contracting path that extracts sufficient semantic context from *D*∘*U* ^*n*^ and a symmetric expanding path that produces the up-sampled output. The contracting path starts with two applications of 3 × 3 convolutions, each followed by a batch normalization layer and a ReLU operation. Then a 2 × 2 max-pooling operation is performed for down-sampling where the number of feature channels is doubled. Then another two 3 × 3 convolutions operations are applied, each followed by batch normalization and a ReLU activation. The expanding path consists of an up-sampling of the feature map, followed by two 3 × 3 convolutional layers, again with batch normalization and ReLU operations. Finally, a 1 × 1 convolution is applied to map the 16-component feature to a single feature channel that reconstructs the transformed image corresponding to N_F_(*D U* ^*n*^).

### Patient Data and Network Training

In this work, 64 eligible oropharyngeal cancer patients who received curative-intent IMRT in our department were retrospectively studied under an institutional review board (IRB)-approved ^18^FDG-PET imaging study (21). All patients were prescribed 70 Gy at 2 Gy/day fraction with concurrent chemotherapy. Prior to treatment initiation, each patient underwent an ^18^FDG-PET/CT scan for target delineation. After 20-Gy delivery, each patient underwent a second ^18^FDG-PET/CT scan as an intra-treatment evaluation for consideration for adaptive planning. These post-20-Gy ^18^FDG-PET acquisitions were treated as the post-radiation scans in the modeling.

All ^18^FDG-PET/CT exams were acquired by a PET/CT scanner (Siemens, Erlangen, Germany) in our department. PET acquisitions were performed using 400 × 400 matrix size in a standard field of view (FOV) of 54 cm, and slice thickness was 2 mm. CT acquisitions were performed using 512 × 512 matrix size in an extended FOV of 65 cm (in-plane resolution = 1.27 mm), and slice thickness was 3 mm. PET images were reconstructed by the ordered subset expectation maximization (OSEM) algorithm with attenuation corrections using the CT acquisition information. The post-20-Gy ^18^FDG-PET/CT images were registered to the corresponding pre-radiation images using Velocity™ software (Varian Medical Systems, Palo Alto, CA, USA). Registrations started with rigid bony structure alignment, and a multi-pass deformable registration algorithm was adopted to improve soft tissue alignment near the anterior body surface. In the process of IMRT planning, all treatment plans were optimized and calculated using the Eclipse™ treatment planning system (Varian Medical Systems, Palo Alto, CA, USA) with a 2.5-mm dose calculation grid size. All ^18^FDG-PET images and spatial dose distribution maps of 20-Gy treatment were resampled to the CT simulation image grid size.

Of all 2D ^18^FDG-PET axial images, those with sufficient ^18^FDG uptake in the pre-radiation acquisition were selected by SUV_max_ > 1.5 excluding brain regions (22). Overall, 718 axial slices collected from 38 patients were used for neural network training, 233 axial slices from 13 patients were used for validation, and 230 axial slices from 13 patients were used for independent tests. During the neural network training, the loss function was defined as based on the *l*_2_ norm in Eq. (8). Gradient updates were computed using batch sizes of 10 samples, and batch normalization was performed after each convolutional layer. The training utilized the Adam optimizer for up to 400 epochs, while an early stopping strategy on the loss function evaluated on the validation samples was adopted with a patience of 100 epochs. The overall training time was about 15 min in a TensorFlow environment using an NVIDIA TITAN™ Xp graphic card.

### Evaluation

The accuracy of post-20-Gy ^18^FDG-PET image prediction was evaluated using 230 axial slices’ results from 13 test patients. The prediction results were first visually inspected as qualitative evaluation. SUV mean values in high-uptake regions determined by Otsu’s method (23) were quantitatively compared with the ground-truth results. Pixel-to-pixel SUV numerical differences were evaluated by Gamma tests within the body region (24). Multiple Gamma tests with different SUV difference tolerances and distance-to-agreement (DTA) tolerances were performed. While Gamma Index <1 was considered as acceptable pixel-wise results, Gamma Index passing rates, i.e., the percentage of pixels with Gamma Index <1, were reported as summarizing metrics.

## Results

**Figure 2:**
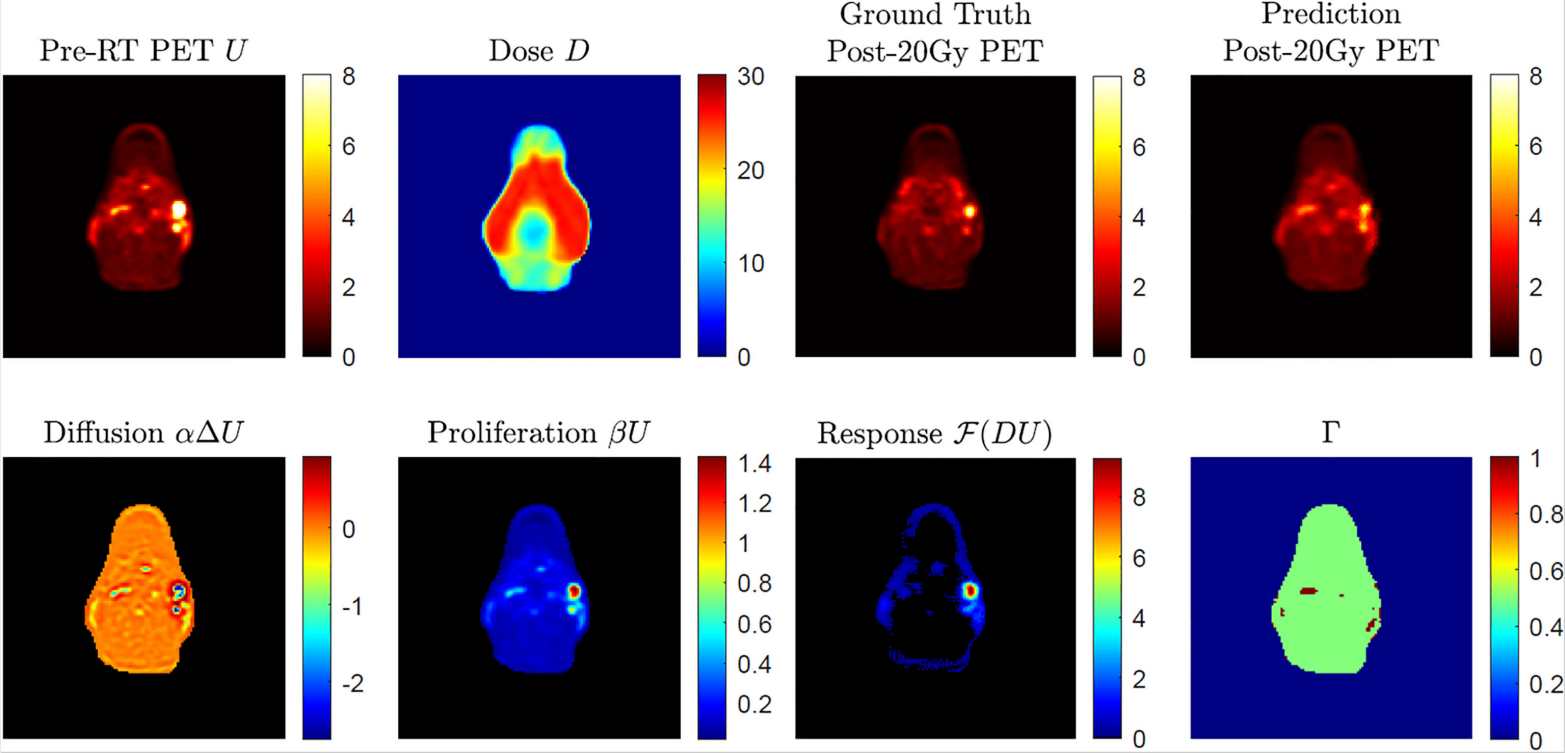
An example of post-radiotherapy ^18^FDG-PET image outcome with given pre-radiation ^18^FDG-PET image and dose distribution map D, with a breakdown of predicted biological effects (diffusion, proliferation, and dose response in absolute value) in Eq. (2). The 2D Gamma (Γ) test result was obtained through acceptance criteria of 5%/10 mm.

**Figure 2** shows an example case of post-20-Gy ^18^FDG-PET image outcome prediction. As seen in the pre-radiation ^18^FDG-PET image, SUV hotspots with clear edges were found on the patient’s right side. After 20-Gy delivery shown by the bilateral side dose distribution in *D*, the ground-truth post-20-Gy^18^FDG-PET image results demonstrated good therapy response with reduced hotspot sizes and decreased SUV intensities. The predicted ^18^FDG-PET image captured the overall appearance in the ground-truth results without noticeable artifact marks. Two hotspots corresponding with the nodal disease were found in the prediction image at the same locations. The hotspots’ sizes and SUV intensities were comparable, though the anterior hotspot intensity appeared to be lower than the ground-truth result. In the breakdown illustration of biological model terms in Eq. (3), the diffusion term demonstrated overall uniform intensity distribution around 0 except in hotspot regions; the core regions in hotspots had negative diffusion intensities, which suggested a spatial retraction of abnormal metabolism. The proliferation term had a similar appearance to the pre-radiation ^18^FDG-PET image. The dose-response term indicated an elevated intensity region that corresponds to the anterior SUV hotspot; this suggests that the anterior SUV hotspot had a better response to 20-Gy than the posterior SUV hotspot, which had limited intensity in the dose-response map. The other areas within the body had close-to-zero dose-response intensity, while low intensities were found near the body surface. The Gamma Index map showed a good quantitative pixel-to-pixel SUV comparison between ground-truth and predicted post-20-Gy ^18^FDG-PET images using the 5%/10 mm criterion.

**Figure 3:**
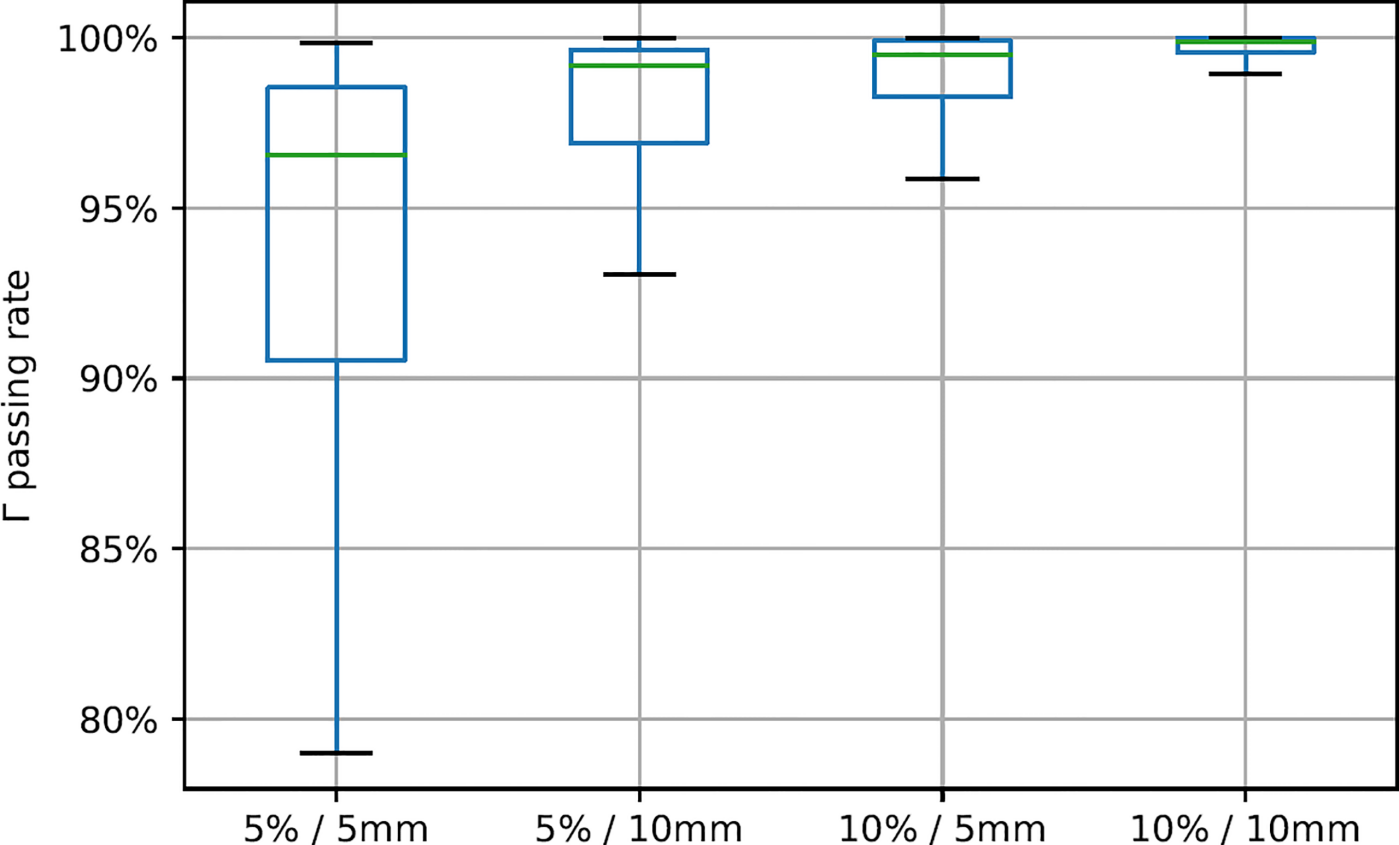
Gamma Index passing rate summary with different gamma criteria. Green line positions represent median value, and box represents 25%/75% percentile with whiskers indicating 5%/95% percentile.

In the test patient cohort, the SUV mean value of high-uptake regions in post-20-Gy predicted images was 2.45 ± 0.25, which was slightly lower than ground-truth results (2.51 ± 0.33, *p* = 0.015); the dice coefficient results of the segmented high-uptake regions were 0.89 ± 0.12. Gamma Index passing rate results of all testing axial slices are summarized in **Figure 3**. When the 5%/5 mm Gamma criterion was adopted, the median 2D Gamma passing rate was 96.5%. With the use of looser Gamma criteria, the passing rate results improved (5%/10 mm, median 99.2%, average 97.6%; 10%/5 mm, median 99.5%, average 98.6%). The highest median passing rate was 99.9% (average = 99.6%) when 10%/10 mm was used.

## Discussion

In this work, we successfully demonstrated the design of a biological model-guided deep learning framework for post-20-Gy ^18^FDG-PET image outcome prediction in a unique cohort of patients undergoing IMRT for oropharyngeal cancer. For the first time, we demonstrated 3 breakdown biological components of oropharyngeal cancer response to radiation. One of the key innovations in this work is the biological model in Eq. (2), which was hypothesized as the mathematical equation that governs the post-radiation SUV change. The model was derived from the classic reaction–diffusion system, which has been utilized in many works of tumor growth and disease progression modeling (25–27). Although applying reaction–diffusion models to ^18^FDG-PET image analysis (particularly to head and neck cancer) is less reported, some exploratory studies have demonstrated the validation of reaction–diffusion-type models in intracranial PET image modeling (28). Compared to the original reaction–diffusion models, the newly introduced dose-response term in Eq. (2) was hypothesized as a semantic component of dose-induced SUV image state changes. Adding additional terms in reaction–diffusion family models to account for therapeutic effect has been reported before in breast, lung, and pancreatic cancer studies (29–31); nevertheless, our approach of using spatial dose distribution in biological modeling is a novel design. Compared to the use of prescription dose levels for outcome assessment/prediction in many studies, the adoption of spatial dose distribution maintained heterogeneous radiation deposition information at the pixel level, which may be a more accurate approach for image-based outcome prediction with explainability from existing biology domain knowledge. Nevertheless, the designed deep learning model relies on the reaction–diffusion system hypothesis, which has yet to be widely acknowledged as general domain knowledge of tissue radiation response. In addition to the image result supports, the reaction–diffusion system hypothesis can be studied *via in vivo* functional imaging (such as diffusion-weighted MRI) and *in vitro* cell study to establish the benchmark evidence for oropharyngeal cancer applications.

As a deep learning approach, a CNN was designed to learn the proposed biological model. PDEs with known coefficients and operators can be solved by various numerical methods such as finite difference methods, finite element methods, and spectral methods. In the scientific computing field, solving differential equations using CNN in complex systems has become popular for efficiency and accuracy (32). Additionally, differential equations specified by CNN can parameterize the continuous state transition with non-uniform sampling step sizes (33); that is, one may use images from different patients with different acquisition time points. The applied analysis of stochastic differential equations has demonstrated value for recent radiomic applications (34, 35); deep learning-based data assimilation may improve the performance of these techniques by providing a more accurate estimation of model hyper-parameters and coefficients. The use of CNN is necessary to learn the dose-response term F in Eq. (2), which is an unknown operator that is assumed to be related to the product of spatial dose distribution and ^18^FDG-PET image variable (*DU*); without an analytical expression, it is difficult to approximate the operator F by classic numerical treatments of inverse problems. The proposed CNN in **Figure 1** revealed the dose-response term F(*DU*) as a whole, while the detailed mechanism of DU‘s contribution of ^18^FDG-PET image prediction remains unclear. Inspired by the classic encoder–decoder U-net implementation, the CNN architecture in **Figure 1** was dedicated to the problem in Eqs. (3)–(7); with the loss function defined in Eq. (8), the training process had a fast convergence (**Supplementary Figure 1**). It would be of interest to further study the operator F for its analytical expression and possible biological explanations. Such works require more advanced mathematical theories supported by experimental data, preferably as *in vitro* implementations, to validate analytical designs as a biological model calibration process (10).

Based on the Gamma test results in **Figure 3**, the achieved ^18^FDG-PET image predictions showed good agreement with ground-truth images. As a common quality assurance method in radiotherapy practice, Gamma analysis accounts for both intensity differences and systematic shifts in image prediction error. The Gamma test criteria need to consider multiple uncertainty sources in data processing and clinical preferences. For instance, the dose-response term results in **Figure 2** indicated very small but non-zero intensity values near the body surface, especially in anterior skin regions. While other normal tissues demonstrated very limited dose response, the observed skin regions’ dose response may be noisy results related to deformable image registration uncertainties, which was mainly determined by patient weight loss during the radiotherapy course (36). Radiotherapy margin formulism that models treatment margin statistics should also be weighted in image prediction evaluation (37). In addition to these two potential factors, the adopted Gamma test criteria have incorporated many other factors, including SUV’s intrinsic uncertainty, PET image acquisition resolution, PET-CT QA protocol, and SUV-based metabolic volume definition.

**Figure 4:**
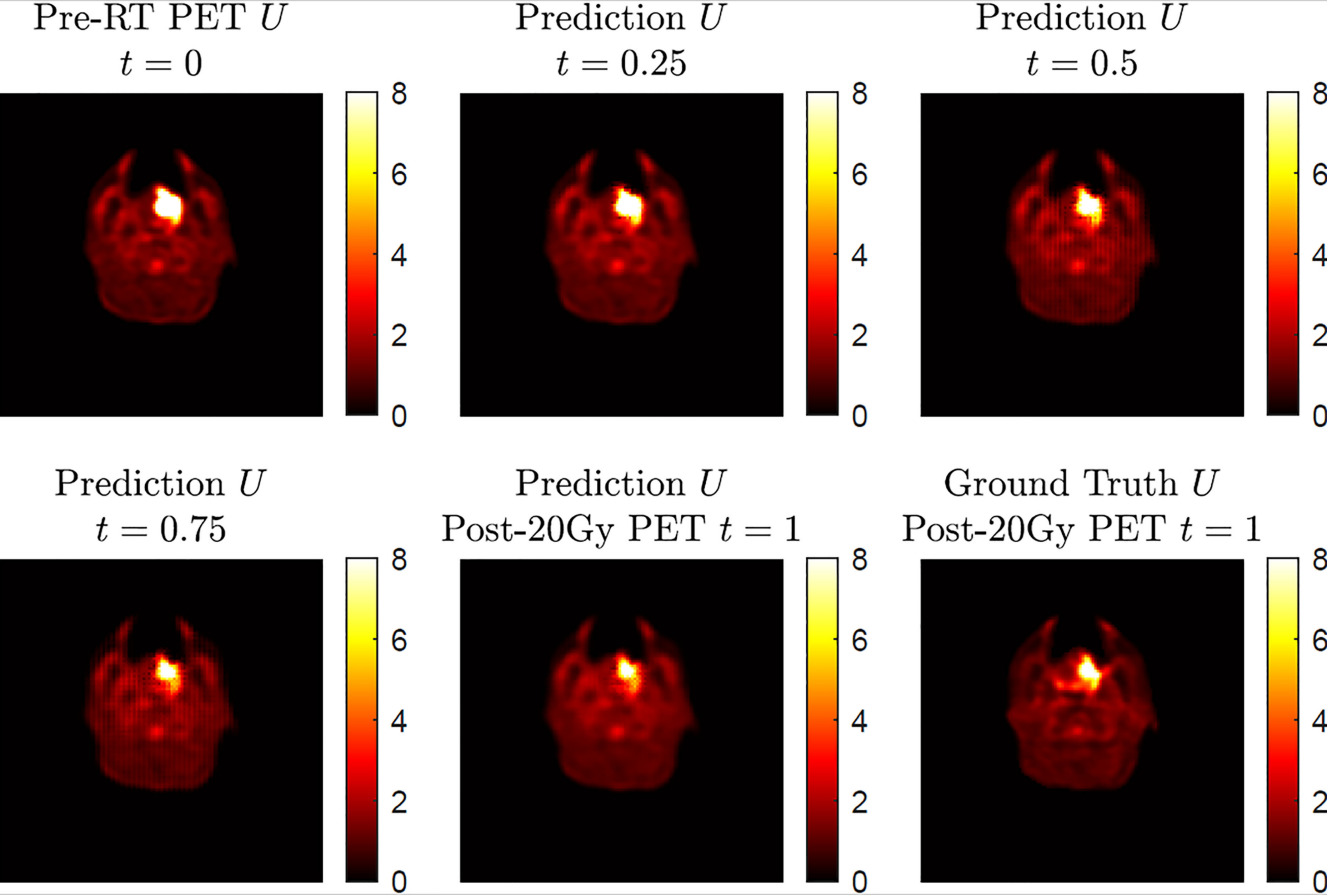
A simulation of ^18^FDG-PET image outcome transition based on a four-step execution with step size *h* = 0.25 showing the predicted transition from pre-radiation *U* at *t* = 0 to the predicted post-radiotherapy *U* at *t* = 1. The ground-truth post-20-Gy ^18^FDG-PET image is included for comparison at *t* = 1.

The current results demonstrated accurate image outcome prediction at the time point of post-20-Gy radiotherapy. The actual physiological change during the 20-Gy radiotherapy course is a continuous process, which is an inherent feature in the proposed model in Eq. (2); in other words, in addition to post-20-Gy ^18^FDG-PET image outcomes at *t* = 1, our model can predict intermediate stage image outcomes between *t* = 0 and *t* = 1. To demonstrate this merit, **Figure 4** shows a simulation of intermediate stage ^18^FDG-PET image outcome predictions as biological model solutions from the pre-radiation result at *t* = 0 to post-20-Gy prediction at *t* = 1. In general, the four predicted ^18^FDG-PET images demonstrated a reasonable image state transition from *t* = 0 to *t* = 1 without abrupt changes. While the majority of normal tissue maintained steady SUV intensities during the presented time evolution, the SUV hotspot corresponding to the primary oropharyngeal tumor had shrinkage at its posterior boundary with slightly reduced intensity. Compared to the ground-truth post-20-Gy ^18^FDG-PET image, the prediction image at *t* = 1 captured the SUV hotspot’s morphological features, particularly at its posterior boundary. However, this simulation result cannot be validated by current clinical results because of the lack of longitudinal ^18^FDG-PET scans during a radiotherapy course, which is mainly limited by ionizing radiation risk and potential high financial cost. On the other hand, longitudinal MRI exams are commonly utilized for cranial radiotherapy follow-up as standard care, and the image series can be used to validate the cranial model continuity in future works.

The current biological model was implemented in a 2D fashion on axial images. For each test patient, the post-radiation ^18^FDG-PET image predictions were generated slice-by-slice to approximate volumetric rendering. In theory, the biological model in Eq. (2) and the demonstrated deep neural network could be implemented as 3D in the spatial domain; however, the computation workload for 3D implementation, especially for a generative task with complex nature, requires a large data sample size with curated data collection. In this work, 64 patients were collected with paired ^18^FDG-PET exams in a clinical trial setup, and 1,181 high ^18^FDG uptake axial slices were collected and were assigned to neural network training/validation/tests following a 60%/20%/20% ratio. Given the fact that 1) image slice thickness (3 mm) is larger than in-plane resolution (1.27 mm) and 2) paired image acquisitions were performed with a 2-week time interval, the model was confined for locoregional computation with a small 3 × 3 in-plane kernel size, and thus the information extraction was within an axial “slab” and did not involve information exchange in other slices. This underlying design made all 2D slices eligible as independent samples for deep learning training, and the current results from 2D implementation demonstrated good image prediction accuracy and established the technical feasibility of the proposed biological model-guided deep learning. 3D-based modeling would be ideal for brainstorming experiments, but this data cohort would be a very limited data sample size for generative deep learning tasks. Future studies using a larger patient cohort, potentially in a multi-institution collaboration, are planned to further investigate the proposed framework based on 3D implementation. Additionally, experiments using small animals are also planned for future developments of deep learning in image outcome prediction. Further investigation of the biological interpretation of the learned dose-response term may also lead to improved mathematical modeling for this problem.

As a feasibility study, the current results showed that the achieved post-20-Gy ^18^FDG-PET image outcome prediction had good agreement with ground-truth results. Post-20-Gy ^18^FDG-PET has been demonstrated as informing surrogates of recurrence-free survival and overall survival of human papillomavirus (HPV)-related oropharyngeal cancer (38). In a potential clinical application scenario, the current framework would allow a physician to determine if an ^18^FDG-PET scan after 20-Gy radiation would facilitate improved adaptive radiotherapy clinical decision making. The impact of image prediction accuracy on clinical decision making was not rendered by the current results of technical development work; future works, preferably in a prospective fashion, are planned to investigate such clinical impacts from physicians’ perspectives in clinical practice. Another crucial step toward this clinical application scenario is to verify the models’ responses to different radiation therapy strategies. The current patient cohort from a clinical study received a uniform treatment regimen; thus, the developed model may not capture certain individual reactions after a drastically different radiotherapy approach. For deep learning developments, it would be ethically challenging to collect patient data with intentional treatment variations. Following the small animal experiments discussed above, with dedicated imaging platforms and radiotherapy machines, one can generate post-radiation samples with heterogeneous treatment strategies in multiple imaging sessions. Such experiments may provide valuable opportunities for studying biological models for improved deep learning intelligibility.

## Conclusion

In this work, we developed a biological model-guided deep learning method for post-radiation ^18^FDG-PET image outcome prediction. The proposed biological model incorporates spatial radiation dose distribution as a patient-specific variable, and a novel CNN architecture was implemented to predict post-radiotherapy ^18^FDG-PET images from pre-radiation results. Current results demonstrate good agreements between post-20-Gy predictions and ground-truth results in a cohort of patients with oropharyngeal cancer. Future developments of the current methodology design will enhance the applicability of image outcome prediction in clinical practice.

## Data Availability Statement

The datasets studied for this study is collected from a clinical trial; due to PHI protection, the original image data cannot be published in the public domain. The studied deep learning design model will be available upon direct request to the corresponding author. Requests to access the datasets should be directed to chunhao.wang@duke.edu.

## Ethics Statement

The studies involving human participants were reviewed and approved by Duke University. The patients/participants provided their written informed consent to participate in this study.

## Author Contributions

All authors participated in the study design. KL and CW collected image data. HJ completed computation works. All authors participated in writing and approved the final version.

## Funding

HJ and AB were supported by the Simons Foundation Math+X investigator award number 510776 and the National Science Foundation under grant NSF DMS-1952339 during this work.

## Conflict of Interest

The authors declare that the research was conducted in the absence of any commercial or financial relationships that could be construed as a potential conflict of interest.

## Publisher’s Note

All claims expressed in this article are solely those of the authors and do not necessarily represent those of their affiliated organizations, or those of the publisher, the editors and the reviewers. Any product that may be evaluated in this article, or claim that may be made by its manufacturer, is not guaranteed or endorsed by the publisher.
