# Supplementary material for: Post-Radiotherapy PET Image Outcome Prediction by Deep Learning Under Biological Model Guidance: A Feasibility Study of Oropharyngeal Cancer Application
Source: Front Oncol. 2022 May 13;12:895544. doi: 10.3389/fonc.2022.895544 (PMC9135979; doi:10.3389/fonc.2022.895544)
Supplement: Supplementary file 1 [file Table_1.docx]

Supplementary Material

## Supplementary Figures

**Supplementary Figure 1.** Training (blue) and validation (orange) loss history, where the solid curves denote the average loss values over 10 independent runs, and the translucent bands show the 95% confidence interval at given numbers of epochs.
